# Contours of a research ethics and integrity perspective on open science

**DOI:** 10.3389/frma.2023.1052353

**Published:** 2023-05-10

**Authors:** Tom Lindemann, Lisa Häberlein

**Affiliations:** European Network of Research Ethics Committees (EUREC), Bonn, Germany

**Keywords:** open science, research ethics, research integrity, responsible research, responsible conduct of research, research governance, ethical reflection

## Abstract

This article argues that adopting a research ethics and integrity perspective could support researchers in operationalizing the open science guiding principle “as open as possible, as closed as necessary” in a responsible and context-sensitive manner. To that end, the article points out why the guiding principle as such provides only a limited extent of action-guidance and outlines the practical value of ethical reflection when it comes to translating open science into responsible research practice. The article illustrates how research ethics and integrity considerations may help researchers understand the ethical rationale underpinning open science as well as recognize that limiting openness is necessary or at least normatively permissible in some situations. Finally, the article briefly discusses possible consequences of integrating open science into a responsibility-centered framework and implications on research assessment.

## Introduction

Even though it is debatable to what extent open science constitutes a new way of conducting research or is more plausibly conceptualized as a revival of the ethos of science (Merton, 1973), the rise of information and communication technologies enables an unprecedented degree of openness and transparency that extends far beyond the traditional focus on open access to publications. What is more, societal expectations toward the research system have changed, resulting in a growing demand for transparency and opportunities for participation. As a result of these tendencies, open science has become an umbrella term that subsumes various principles and practices, such as open data, open reproducible research, open science evaluation and open educational resources (Fecher and Friesike, 2014).

Expectations to follow open science recommendations increasingly permeate all segments of the academic system and thus create increasing demands on researchers and the entire research community, not least because major research funding organizations, such as the European Commission and cOAlition S members, increasingly tie funding to following open science practices. As open science practices change research conduct and the relationship between researchers, research participants and society, it has important repercussions on research ethics and integrity. Thus, it is likely that a growing number of researchers will need cognitive and affective knowledge, skills and competencies not only in research ethics and integrity, but also in open science. Understanding how open science and research ethics and integrity are related is important to avoid sending mixed signals to researchers about their normative obligations and, moreover, to ensure research assessment schemes reinforce rather than undermine responsible conduct of research. This article takes initial steps on this path and shows how research ethics and integrity considerations can help pave the way toward responsible open science and briefly outlines how narrative elements in researcher assessment schemes can support the open science transition envisaged by many key actors in the research governance ecosystem.

Since research ethics and integrity and open science differ in their rhetorical orientations, the risk of inadvertently sending mixed signals is by no means trivial. Whereas research ethics and integrity often restrict the realm of legitimate research practices, open science explicitly aims to broaden the notion of good scientific practice by asking researchers to invest time and resources into making their research accessible and transparent. Although this does not imply that research ethics and integrity and open science place contradictory demands on researchers, their different framings can confuse researchers who might feel pressured to simultaneously respond to open science recommendations as well as, for example, seemingly countervailing data protection obligations. Because most researchers are neither experts in open science nor in law, navigating various regulatory regimes and understanding how they are related often creates practical difficulties. At one end of the spectrum, there is a risk that researchers disregard safeguards in the pursuit of open science. At the other end, there is a risk that they fail to open their research due to unwarranted fears of breaching legal or ethical obligations. Navigating between these poles is challenging and requires attention to complexity.

In the remainder, we argue that adopting a research ethics and integrity perspective has the potential to operationalize the open science guiding principle “as open as possible, as closed as necessary” in a practical and responsible manner. We draw on desk research as well as tentative findings from 12 semi-structured qualitative interviews and three focus groups with 17 participants in total conducted in the first half of 2022 as part of the EU Horizon 2020 project “Responsible Open Science in Europe” (ROSiE). The interviews were transcribed and analyzed with respect to the thematic foci of the ROSiE project, namely ethical, legal, social, and integrity aspects of open science and citizen science. Interviewees included researchers from medical and health sciences, social sciences, and the arts and humanities, research integrity officers, research managers, representatives of research funding organizations, policymakers, and science educators. Focus groups participants included five researchers of various career stages (from early career to senior) from the medical and health sciences, social sciences, and engineering and technology, as well as six members of research ethics committees, a representative of a research funding organization, a research manager responsible for research ethics and integrity at a higher education and research performing organization, two research policymakers, a data journalist, a science educator, and a representative of a science engagement organization [for further information, see Lindemann et al. (2022); the interview guide and the focus group guides are available as Supplementary material to this article].

The article does not aim to provide a full analysis of the relationship between research ethics and integrity and open science but attempts to show that an integrated perspective could improve research governance by helping researchers to understand and endorse their normative obligations.

## A pathway toward operationalizing open science

As an interviewee with a background in the humanities pointed out, the term open science can be criticized for being insufficiently inclusive as it inadvertently gives the impression of excluding the arts and humanities from its purview, despite intending to cover all fields of research. We concur that speaking of research rather than science could help clarify misunderstandings about the scope of open science and would indeed signal greater inclusivity, yet our interviews and focus groups suggest that most practical challenges researchers face when trying to implement open science practices are primarily related to operationalizing the meaning of openness. The term open science as such offers little guidance in that regard because it refers to the process of opening research rather than a discernible moral purpose. The guiding principle “as open as possible, as closed as necessary” is more useful in practice as it clearly recognizes that open science refers to openness within limits, yet it does not offer any specific guidance on what these limits are.

Obviously, legal obligations related to, for example, data protection law define clear boundaries to openness so that opening up research is more challenging and complex in, for example, medical and health sciences where the processing of personal data very often is necessary to conduct meaningful research than in, for example, the natural sciences, where personal data processing is not common. However, research is to a significant extent regulated by soft law, such as recommendations, guidelines, standard operating procedures and shared norms that are not explicitly codified. Consequently, it is implausible to assume that only legal obligations can provide legitimate reasons for not opening research. As research ethics and integrity are pillars of existing governance arrangements aimed at ensuring responsible research, they offer several starting points for defining both legitimate boundaries to openness in concrete research settings and underscoring the importance of openness whenever none of the boundaries applies. Because of that, reflecting on the implications of the “as open as possible, as closed as necessary” principle from a research ethics and integrity perspective could help researchers to operationalize open science recommendations in a way tailored to their situation and responsive to as well as compliant with pertinent ethics and integrity norms. Before outlining some key issues meriting consideration in reflection processes, we briefly elaborate the promises of reflection on principles, values and norms for decision-making on open science.

### The practical value of ethical reflection on open science

Acquiring an accurate understanding about what open science means in their field of research and in their concrete situation and the capacity to decipher mixed signals is crucial for the ability of researchers to follow and endorse open science practices in a conscious and responsible manner. This requires tailored upskilling and reinforcing good research practice through normatively appropriate reward and recognition systems. While research institutions play a key role in empowering researchers by providing access to infrastructures, offering training and rewarding and incentivizing good practice, researchers themselves can cultivate habits conducive to responsibly navigating complex environments. Ethical reflection is an effective mechanism to foster such cultivation and helps researchers to competently weigh different arguments in morally challenging situations and resolve or mitigate contradictions in a justifiable way (see Evans et al., 2023).

According to Dutilh Novaes (2022), reflection is an epistemic practice that involves the critical examination of reasons for and against a practice under scrutiny with the aim of arriving at stronger and more justified beliefs. Consequently, reflection is particularly useful if actors are confronted with overtly contradictory norms. Reflections should be open and consider all relevant perspectives, including arguments that could affect and change entrenched beliefs about good practices (Dutilh Novaes, 2022). Hence, thorough and open reflection can revise convictions and ultimately reorient conduct. Applied to the realm of open science, ethical reflection can shape the beliefs of researchers and increase their knowledge and understanding of how “as open as possible, as closed as necessary” and other guiding principles can be harnessed for orientation.

A metaphor might help to illustrate the process of weighing different arguments in favor of and against opening a given piece of research: Think of open science as a door hinge that allows both opening and closing the door. Depending on what researchers choose to do, they open or close the door to certain information. Whether the door should be open, closed or ajar depends on the situation and cannot easily be answered in the abstract, not least because the degree to which research environments are supportive of open science varies considerably. Researchers based in the scientific center, for example, usually are significantly better positioned to follow open science practices than researchers in the periphery because of better access to cutting-edge research infrastructures, as one of our focus groups highlighted. Such situational factors are difficult to integrate into general guidelines, but they nonetheless have clear moral significance. Reflecting on the characteristics of a given situation through an ethics and integrity prism often can help researchers decide how far to open or close the metaphorical door and thus empower them to competently navigate challenges they might encounter in their daily research practice.

### Research ethics, research integrity and open science

Research ethics and research integrity overlap in various ways. By and large research ethics refers to the study of moral problems related to research and focuses on the relationship between the research system on the one hand and research participants as well as human society and the environment on the other. Research integrity, by contrast, refers more narrowly to good scientific practice and conduct within the scientific community (Steneck, 2006, p. 55–56). Overall, research ethics and integrity are based on complementary and partly overlapping values and principles, such as justice, beneficence, non-maleficence, respect, accountability, reliability, and honesty. Together, they form a central pillar of existing research governance schemes and are a major source of normative expectations researchers are expected to meet.

We argue that a research ethics and integrity perspective on open science is pragmatically useful in helping researchers to recognize the value of openness as well as to understand when openness should be restricted. This perspective emphasizes the importance of situational reflection in translating abstract principles into concrete action recommendations and therefore avoids the pitfalls of a one-size-fits-all approach that would presumably either be insufficiently ambitious or overburden some researchers. Drawing on key concepts from research ethics and integrity, we illustrate how ethical reflection can help researchers navigate the open science landscape. The following overview of normative arguments to consider when deciding in favor of or against opening research does not aim to cover all potentially relevant considerations. It rather intends to show that a research ethics and integrity prism on open science has added value because it may help researchers understand arguments for and against opening research and help them in navigating tensions in a competent and ethically appropriate manner. Even though we point to disciplinary specifics to some degree when discussing issues related to data protection, we do not aim to address all field-specific nuances of open science. We rather seek to present contours of a possible overarching framework for reflection that, in a next step, could be tailored more systematically to disciplinary and other relevant differences.

#### “As open as possible”: considerations from a research ethics and integrity perspective

Both research ethics and integrity offer many points of reference in favor of opening research. One of the most intuitive ethical arguments for open science is that it is capable of significantly strengthening the relationship between the research system and society by making research results more easily accessible and research more participatory. In other words, open science can help to make research responsive to societal needs and empower actors hitherto at the margins of the scientific endeavor to get actively involved in research and innovation.^1^ Increased participation comes in at least two forms, namely more thorough stakeholder involvement and more citizen or participatory science. Stakeholder engagement as well as citizen participation in research support the alignment of research goals and procedures with societal needs and values and helps making research relevant, even though the potential for tensions with the important principle of academic freedom should not be entirely discarded. Especially proposals for ethics assessment procedures in new and emerging technologies, such as ethics by design approaches, are based on strong stakeholder engagement in different phases of the research process (Brey et al., 2022). These approaches presuppose that research is sufficiently open to function. Therefore, open science and ethics by design coalesce in important ways.

Consequently, research ethics arguments for opening research primarily focus on bolstering the science-society nexus, which is key to ensure the societal relevance and ethical appropriateness of certain types of research. As a result, a research ethics perspective may help researchers recognize the value of openness both from a moral and a relevance standpoint and decrease skepticism toward open science practices. In the same vein, open science has the potential to readjust research conduct to the ethos of science that has historically served as the normative superstructure of the Republic of Science, that is, the system of publicly and philanthropically funded research, of which a relatively high degree of openness has been a hallmark (cf. Merton, 1973; David, 2008).

In addition, there are numerous research integrity-related arguments in favor of open science, and it seems fair to say that researchers aiming to act with integrity should generally take a sympathetic stance toward opening up research. Haven et al. (2022) even argue that open science and research integrity are essentially two sides of the same coin as both strive to make research more traceable and verifiable. Typical research integrity arguments for open science mentioned in our interviews and focus groups include that openness improves accessibility of knowledge to researchers from all over the globe, strengthens reproducibility by enhancing access to research data and research procedures, reduces research waste by enabling access to effective research methods and avoiding unnecessary duplications of failed studies, and increases reliability by facilitating effective review and appraisal of results. Thus, research integrity considerations on the aspirational level clearly favor openness as it has the potential to enhance the capacity of the research system as a whole and thereby increase research quality. However, as the next section will show, the almost complete alignment of research integrity and open science rests on more shaky foundations when researchers are confronted with the question what research integrity means in imperfect environments.

#### “As closed as necessary”: considerations from a research ethics and integrity perspective

Despite offering strong arguments in support of open science, ethical considerations also can be helpful in delineating legitimate limits to openness. The most important arguments brought forward in our interviews and focus groups fall into two broad categories, namely privacy risks and dual use and misuse concerns. Consequently, focal arguments against opening research mostly apply to research with human participants and research with dual-use potential. The governance of privacy risks is closely related to data protection law and thus to a significant extent regulated by legal obligations. However, the protection of the privacy of research participants also is a cornerstone of research ethics. As far as research with human participants is concerned, open science stands in tension with established models of informed consent because traditional consent models require consent to be specific, which is often already challenged by research on, and with, new and emerging technologies, where potential risks are often difficult to determine in advance (see Deutsche Forschungsgemeinschaft, 2019, p. 73). While newer broad and dynamic consent models might mitigate or even solve this problem, concerns about the specificity of consent should not easily be swept aside until a normative consensus on alternative forms of informed consent has emerged.

Another noteworthy privacy risk is related to the effectiveness of anonymization techniques. According to the GDPR, anonymous data is not personal data and therefore not subject to data protection law. Yet technological advancements in artificial intelligence and big data research and analytics might enable deanonymization and profiling on an unprecedented scale through linking information from various datasets, as a research ethics committee member emphasized in a focus group. Consequently, researchers intending to share anonymized data should assess whether the anonymization technique they have used is as robust as possible and what the likely consequences of deanonymization would be for data subjects. While such concerns should not be used as a carte blanche for refraining from practicing open science in research involving personal data, it would be equally false to ignore them. Close attention should be paid to the thoroughness of the reasoning process and it should be recognized that giving unambiguously correct answers might not always be possible due to high amounts of uncertainty.

Besides privacy risks, dual use and misuse concerns should be factored into reflection on whether and to what extent to open up research. The ethical significance of dual use problems is recognized in research governance and regulated by a specific regime that poses limits to open science in the interest of protecting human society and the environment. As a result, dual use and misuse concerns attenuate the societal desirability of openness. In addition to traditional dual use and misuse concerns, open science exacerbates risks related to inadvertent misuse due to incompetency. Although improved access to research results, data and methods offers many promises, it also enables people who lack the necessary competencies and access to research infrastructures to try to conduct potentially risky research. Considerations related to risks of unregulated do-it-yourself research thus should not be entirely discarded, although it is difficult to assess their magnitude and important to ensure that they are not abused as a carte blanche for restricting openness.

Research integrity-based reasons for limiting openness are mainly related to the moral effects of acting in imperfect research environments. Notwithstanding the fact that the aspirations of research integrity and open science are complementary, acting with integrity in challenging circumstances might pose justifiable limits to openness. Many interviewees and focus group discussants, confirming a major finding of Laine (2017), cited the widespread fear of scooping as a reason for the reluctance of many researchers to fully embrace open science practices. Unless research support, assessment and reward systems mitigate this concern by aligning incentive structures to open science and ensuring access to proper research infrastructures also for researchers in the scientific periphery, it should not be dismissed as morally insignificant, although researchers should reflect on the magnitude of the risk of being scooped in their field of research to avoid being misguided by unwarranted worries.

A different type of integrity problem might arise in research conducted at the intersection of academic and industry research because the “Republic of Science” and the “Realm of Innovation” are governed by distinct norms, and openness is only recognized as core value in the former (David, 2008). Therefore, researchers working at the intersection of both regimes might be confronted with partially incompatible normative expectations that, on the one hand, are based on open science recommendations, but emphasize intellectual property rights, trade secrets and other commercial norms on the other. For as long as these different sets of norms remain insufficiently aligned, weighing different arguments and navigating this landscape is challenging so that demanding openness without attention to context seems misplaced.

## Discussion

In order to practice open science, researchers must operationalize the guiding principle “as open as possible, as closed as necessary” for their own research. We have argued that a research ethics and integrity perspective can be useful for doing so because it helps researchers understand both why open science is ethically desirable in general and why under certain circumstances limits to openness are necessary and justifiable. Because the perspective takes into account that many research environments are imperfect and that researchers often find themselves in situations where they need to balance open science with other goods, it avoids the pitfalls of contextually insensitive one-size-fits-all approaches. Moreover, a perspective that recognizes that open science recommendations need to be contextualized and subsumes them under the overarching concept of responsibility in research may have the potential to increase the attractiveness of endorsing open science among researchers hitherto skeptical of its merits.

Our line of argument yields an important implication for research assessment. If the degree of obligatoriness underpinning demands to practice open science depends partly on situational intricacies, quantitative metrics alone are insufficient to measure whether researchers follow open science recommendations in a responsible manner. Thus, our main argument underscores the value of research assessment schemes that include narrative elements and offer researchers the possibility to explain and justify their conduct.

## Data availability statement

The original contributions presented in the study are included in the article/Supplementary material, further inquiries can be directed to the corresponding author.

## Ethics statement

Ethical review and approval was not required for the study on human participants in accordance with the local legislation and institutional requirements. The patients/participants provided their written informed consent to participate in this study.

## Author contributions

TL wrote the manuscript with contributions from LH and both authors approved the manuscript before submission. All authors conceived and designed the study, and prepared, conducted and analyzed interviews, and focus groups as well as relevant literature.

## Footnotes

^1^See Giannelos et al. (2022) on ethics and participation in research and innovation and Penders et al. (2018) on the purpose and functioning of research ethics more generally.
