# Supplementary material for: Contours of a research ethics and integrity perspective on open science
Source: Front Res Metr Anal. 2023 May 10;8:1052353. doi: 10.3389/frma.2023.1052353 (PMC10206019; doi:10.3389/frma.2023.1052353)
Supplement: Supplementary file 1 [file Data_Sheet_1.docx]

# Semi structured focus group guides

## Focus group 1

**Objectives and desired outcomes**

1. Explore research ethics and integrity challenges different stakeholders face when aiming to pursue open science practices.
2. Identify obstacles that make it difficult to realize the transformative potential of open science.
3. Gain insights that might help to develop tools and guidelines to support stakeholders in implementing responsible open science practices.

**Introduction**

The moderator starts the session with basic information about the ROSIE project, situates the focus group discussion in project activities with relevant stakeholders and explains what the focus group interview is about, who is running it what the results of the discussion will be used for. The moderator guides participants through the methods and techniques used in the focus group interview and provides assistance. Participants are welcomed and requested to introduce themselves briefly.

**Method**

My personal business card

**Goal**

Get to know each other, reduce insecurities, and establish a connection to the topic of open science.

**Instruction**

Use a virtual whiteboard. Write down your name, your institution, and your background to create your personal business card. To get to know each other better, complete phrases like:

“I consider the relevance of open science to be ...”

“In my work, open science issues are relevant, in that…”

“I am interested in the topic of open science, in that ...”

**Start the conversation**

**Method**

Three corners method

**Goal**

Start a conversation and identify different opinions and views.

**Instruction**

Assign yourself to one of the following statements:

“I view open science rather as a promise.”

“I view open science rather as a problem.”

“I view open science as both, promise and problem.”

Exchange views and identify similarities as well as differences with other stakeholders. Explain why you have assigned yourself to the relevant statement and share your view with the plenum.

**Moderator questions/probes**

- For whom is open science a promise/problem?
- When you put yourself in the shoes of …, what, if anything, is the promise of open science?
- Can you explain the reasons why you view open science as a problem/promise?
- When you think about the argument that have just been made in the discussion, can you identify sources underlying disagreements?

**Generate input on responsible open science**

**Method**

World Café

**Goal**

Promote a creative approach, generate input, share knowledge, stimulate innovative thinking, and explore action to a defined topic concerning open science.

**Instruction**

Participants discuss a given topic as if they were sitting around a table in a café. They note their results on a virtual white board so that everyone can see them. The discussion should last about 20 minutes.

- In your view, what are the values of responsible open science? Or, asked differently, what values does open science have to endorse to be considered responsible?
- If you think about a scenario of responsible open science, what would be its core features?
  - What about open access/data/educational resources/government?
- How do these values relate to the principles of research ethics and research integrity?
- Can you anticipate any tensions between norms? Where will these tensions play out? Who could suffer loses?
- Can you anticipate new ethical challenges for research ethics and research integrity that are either created or reinforced in an open science context? And who is/will be affected by them?
  - - - - Are these challenges primarily technical or normative? Or both?
        - Are challenges also related to policy, education etc.?

**Close the session**

The moderator closes the session with final remarks and mentions upcoming focus groups that will go on working with the results of today´s interview, trying to explore possible trajectories of open science practices and to identify potential pathways for addressing research ethics and integrity challenges. The moderator cordially thanks all participants for their attendance and contributions.

## Focus group 2

**Objectives and desired outcomes:**

1. Explore possible trajectories of open science practices.
2. Identify potential pathways for addressing research ethics and integrity challenges in a manner conducive to the responsible conduct of research as well as to linking science and society.
3. Use findings to develop tools and guidelines that can support stakeholders in implementing responsible open science practices.

**Introduction**

The moderator starts the session with basic information about the ROSIE project, situates the focus group discussion in project activities with relevant stakeholders and explains what the focus group interview is about, who is running it what the results of the discussion will be used for. The moderator guides participants through the methods and techniques used in the focus group interview and provides assistance. Participants are welcomed and requested to introduce themselves briefly.

**Methods**

My personal business card

**Goal**

Get to know each other, reduce insecurities, and establish a connection to the topic of open science.

**Instruction**

Give a short introduction of yourself. Imagine exchanging business cards with each other. What important information would you like to share about yourself to get to know each other better. Also think about how you would complete sentences like this:

“My thoughts on open science are…”

“When I hear open science, I think about…”

The moderator encourages participants not to hesitate to ask questions, exchange, and gain insights into the fields of interest of other stakeholders.

**Opinions on values and challenges on responsible open science**

**Method**

Flashlight

**Goal**

Improving communication. Opinions on values and challenges identified during FG1 can be expressed spontaneously and briefly without commenting on them. Only questions of understanding are allowed.

**Instruction**

The moderator summarizes the values identified during focus group 1 and initiates a discussion on whether participants agree on their importance. Participants express their opinion in first-person statements on whether they agree on the importance of the values identified during focus group 1. Each person voices no more than one or two sentences or uses emoji responses in zoom to express themselves.

The moderator shares Power Point slides and puts the mentioned values into the chat:

- Research integrity values from the European Code of Conduct for Research Integrity: Reliability, honesty, respect, accountability
- Transparency (highlighted several times)
- Respect (specified as respect also for data)
- FAIR principles: Findability, accessibility, interoperability, reusability (as a legitimate standard)

**Linking challenges to solution, taking promotion of values into account**

**Method**

Collaborative mind map

**Goal**

Encourage creative working techniques to bring together potential pathways for addressing research ethics and integrity challenges from the focus group, to associate and collect them and to identify and link different aspects.

**Instruction**

In 15 minutes per challenge, participants share thoughts, ideas, and contexts with the group without a strict structure. They are encouraged to consider the before mentioned values in the solution finding process.

The moderator leads to the problem areas mentioned in focus group 1 and, if necessary, asks intermediate questions.

1. Lack of standards (As a result of underdetermination of FAIR standards?)

- Lack of quality control
- Lack of training
- Lack of research results publication as a process

How could standards be established? What should they look like?

1. Lack of funding (Is OS currently pursued in an overly ad hoc way, without structural support?)

- Lack of time
- Shortage of staff
- Inadequateness of project applications

What could an infrastructure look like that would guarantee adherence to and promotion of responsible OS?

1. Lack of incentives (Even if doing responsible open science is possible, is it adequately rewarded?)

- Lack of rewards
- Fear of being scooped: How can incentives be created that encourage openness and deter inappropriate scooping?

What should these look like to ensure adherence to standards and funding infrastructure?

1. Inadequate political support structures (Could/should open science practices be elevated on the political agenda?)

- Lack of societal awareness, little incentives for politicians to endorse change.
- Insufficient inclusion of the research community in standard setting/ shaping policy

What could the continuous involvement of researchers and stakeholders look like? How could more OS be promoted in the Global South?

Is an important challenge missing? Are the named challenges real and important?

**Closing the session**

The moderator closes the session with final remarks and mentions the upcoming focus group that will go on working with the results of today´s interview, trying to explore ethical challenges in more depth and identify potential mitigation measures. The moderator cordially thanks all participants for their attendance and contributions and asks about key messages from the focus group discussion. What would be their most important recommendation for the ROSiE project?

## Focus group 3

**Objectives and desired outcomes:**

1. Explore ethical challenges of open science in more depth.
2. Identify possible mitigation measures to realize the transformative potential of open science.
3. Gain insights that might help to develop guidelines on responsible open science, policy advice on how to align the research ethics and open science agendas and training materials for students and researchers.

**Introduction**

The moderator starts the session with basic information about the ROSIE project, situates the focus group discussion in project activities with relevant stakeholders and explains what the focus group interview is about, who is running it what the results of the discussion will be used for. Participants are welcomed and requested to introduce themselves briefly.

**Open science and the ethos of science**:

- How does open science relate to a key set of norms (Mertonian norms) that are often also referred to as the ethos of science (communism, universalism, disinterestedness, and organized skepticism)?
- In your view, can open science also be associated with counter-norms such as secrecy, particularism, interestedness, or dogmatism?
- To what extent can open science strengthen the nexus between science and society?

**Open science and principles of research ethics and ethics review practices**:

- How does open science relate to the key principles of research ethics, such as autonomy, beneficence, non-maleficence, and justice? How can these principles be challenged or upheld by open science practices?
- Does the evolving transition to open science affect the ethics review process? If so, how?
- Do you see any tensions between open science and, for example, privacy and data protection? If so, what are the tensions and how could they be addressed? Have you come across any other challenges related to open science in ethics reviews?

**Closing the session**

The moderator closes the session with final remarks and asks participants about key messages from the focus group discussion. What would be their most important recommendation for the ROSiE project? The moderator cordially thanks all participants for their attendance and contributions.
