# Supplementary material for: Contours of a research ethics and integrity perspective on open science
Source: Front Res Metr Anal. 2023 May 10;8:1052353. doi: 10.3389/frma.2023.1052353 (PMC10206019; doi:10.3389/frma.2023.1052353)
Supplement: Supplementary file 2 [file Data_Sheet_2.docx]

# Semi structed interview guide

*Interviewee:*

*Stakeholder category:*

*Interviewer:*

*Date:*

*Time:*

*Format:*

*Recording:*

## Before the start of the interview

- Collect informed consent
- Introduce yourself and the ROSiE project

## Section 1: Background information and building rapport

- **Can you please tell me about the institution you’re working for? What are the main objectives of the institution?**

*Probes:*

- - When was the institution founded?
  - Have the objectives of the organization shifted over time?
- **What is your current position and what are your main tasks?**

*Probes:*

- - Is that position primarily academic or more related to (research) management?
  - What is your academic background?

| ***Interviewer notes*** |
| --- |

## Section 2: Open science – conceptions and tasks

- **What do you associate with open science?**

*Probes:*

- - What does open science mean to you?
  - Do you view open science rather as a promise or rather as a problem?
- **Are any of your tasks related to open science and, if yes, what are these tasks?**

*Probes:*

- - Which open science issues are most relevant in your work?
  - How often you work on these tasks, and for how long?
  - If answer to initial questions is “no”: Did you have any contact with open science so far? If yes, what kind of contact?
  - Do you have colleagues who work on open science related tasks? If yes, do you know what they’re working on?
- **What, if any, role does open science play for your institution?**

*Probes:*

- Does your institution promote open science and, if yes, how?
- Does your organization promote open science also with technological solutions / normative instruments, like policies and guidelines?
- **In your view, has open science improved the work of your institution or has it rather created problems and challenges? / In your view, could open science help improving the performance of institution or do you think it would rather create new challenges?**

*Probes:*

- - Which aspects of open science have helped you the most?
  - Which aspects of open science have created most problems and challenges?

| ***Interviewer notes*** |
| --- |

## Section 3: Open science, ethics, and integrity

- **Are any of your tasks related to research ethics or research integrity and, if yes, what are these tasks?**

*Probes:*

- - Can you describe these tasks in more detail?
  - Does open science play any role in these tasks? Is open science discussed in your ethics committee/research integrity office/etc.?
- **How would you describe the relationship between open science and research ethics?**

*Probes:*

- - Overall, do you think research ethics and open science are mutually supportive or do you see more pitfalls than promises?
  - How would you address these challenges?
  - How could the promises be realized?
  - Do you see ways how open science could support the work of RECs?
- **How would you describe the relationship between open science and research integrity?**

*Probes:*

- - Overall, do you think research ethics and open science are mutually supportive or do you see more pitfalls than promises?
  - How would you address these challenges?
  - How could the promises be realized?
  - Do you see ways how open science could support the work of RIOs?

| ***Interviewer notes*** |
| --- |

## Section 4: Towards responsible open science

- **What are the main ethical challenges of open science?**

*Probes:*

- - Are these challenges primarily technical or primarily normative? Or both?
  - Are challenges also related to policy, education etc.?

**What strategies do you know to deal with these challenges?**

*Probes:*

How do you think about training in OS?

What added value could training in OS provide?

- - In what ways do you think training in OS could help you to deal with mentioned challenges?
- **What tools could potentially facilitate the move towards open science for you and your institution?**

*Probes:*

- - Could you also benefit from technological / normative tools? If yes, which and how? If no, why not? Are they already in place?
  - If core tools ROSiE will produce are not mentioned: Could you imagine XY being useful for you and your institution?
- **What role do you think stakeholders should play in research?**

*Probes:*

- - Do you think stakeholder engagement improves research or is it rather an obstacle?
- **Are there any examples of good open science practices you would like to share with us?**

*Probes:*

- - Can you explain what it is that makes this practice good? Why has it been so successful?

| ***Interviewer notes*** |
| --- |

## After the interview

- Thank the interviewee
- Inform the interviewee once more about when and how the report on the interviews will be shared
- Take field notes (interviewer notes)
